# Cerebral Infarction Due to Post-traumatic Cerebral Vasospasm in a 12-Year-Old Female

**DOI:** 10.7759/cureus.56275

**Published:** 2024-03-16

**Authors:** Daisuke Tanikawa, Yushiro Take, Nobuaki Naito, Akio Teranishi, Hiroki Kurita

**Affiliations:** 1 Department of Cerebrovascular Surgery, Saitama Medical University International Medical Center, Hidaka, JPN

**Keywords:** post-traumatic cerebral vasospasm, cerebral infarction, ischemic stroke, cerebral vasospasm, diffuse axonal injury, traumatic brain injury, pediatrics, head trauma

## Abstract

Cerebral infarction due to post-traumatic cerebral vasospasm is rare. Although some modalities are recommended to detect post-traumatic cerebral vasospasm, its diagnosis remains controversial and challenging. Therefore, in this report, we will use a case report to highlight challenges and to delineate the characteristics of post-traumatic cerebral vasospasm in pediatric patients, including the diagnostic and treatment options. A 12-year-old female was admitted to our hospital following a motor vehicle collision. Her consciousness was severely impaired. Initial computed tomography (CT) revealed an acute subdural hematoma along the tentorium, and a focal subarachnoid hemorrhage was observed in the Sylvian fissure. The patient underwent the insertion of an intracranial pressure sensor and received therapy for increased intracranial pressure (ICP) control under sedation. On the second day, CT angiography (CTA) revealed no signs of arterial abnormality. A patient who is comatose or under sedation has masked neurological symptoms. Thus, new neurological events could only be detected via an intracranial pressure sensor. Her ICP increased on the seventh day, and a CT scan showed a new cerebral infarction in the right middle cerebral artery (MCA) region. We performed decompressive craniectomy to reduce ICP. Postoperative CTA confirmed severe vasospasm in the right MCA. The severe cerebral vasospasm induced the cerebral infarction. Our review suggests that physicians in trauma departments should frequently perform vascular evaluations by CTA, magnetic resonance angiography (MRA), transcranial Doppler ultrasound, or digital subtraction angiography (DSA), especially within two weeks from onset, to detect post-traumatic cerebral vasospasm.

## Introduction

Various studies have reported and established diagnosis and treatment options for cerebral vasospasm following subarachnoid hemorrhage [1]. Cerebral vasospasm induces ischemic stroke and worsens neurological outcomes [2]. Therefore, it is necessary to detect cerebral vasospasm before cerebral infarction occurs. For patients with subarachnoid hemorrhage, computed tomography angiography (CTA) and magnetic resonance angiography (MRA) should be performed regularly from day 3 to day 14 of onset for the early detection of cerebral vasospasm [1]. However, there are limited evidence and guidelines regarding cerebral vasospasm following head trauma, particularly in children. In a study on 69 children with traumatic brain injury, the rate of vasospasm was 8.5% (Glasgow Coma Scale {GCS} score: 9-12) and 33.5% (Glasgow Coma Scale score: 8 or less) in moderate and severe traumatic brain injury, respectively [3]. Post-traumatic cerebral vasospasm in children tends to develop two to six days after trauma [4]. Although some modalities are recommended for the detection of post-traumatic vasospasm, the diagnosis of post-traumatic cerebral vasospasm is controversial and challenging [5,6]. We report a pediatric case of post-traumatic cerebral vasospasm, including the diagnostic modalities, and suggested options to consider for future use.

## Case presentation

**Figure 1:**
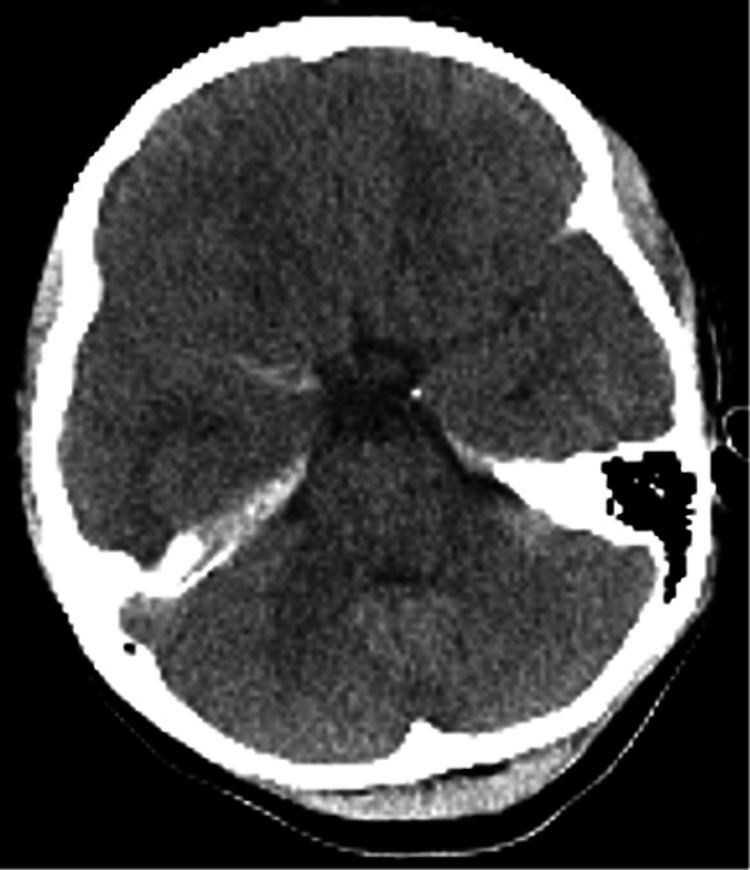
A CT scan of the head on the first day revealed a right temporal lobe cerebral contusion, an acute subdural hematoma in the tentorium, and a traumatic subarachnoid hemorrhage in the Sylvian fissure. No intracerebral hematoma and brain edema were observed. CT: computed tomography

A 12-year-old female was admitted to our hospital after being hit while on her bicycle or perhaps hit and thrown by a car that was going 40 km/hour. Her initial Glasgow Coma Scale (GCS) score was 3. Pupillary examination revealed dilated pupils with diminished light reflex. Her blood pressure increased to 140/50 mmHg, and percutaneous oxygen saturation was 99% with 10 L of oxygen via a nonrebreather mask. An initial computed tomography (CT) of the head revealed a focal acute subdural hematoma along the tentorium and subarachnoid hemorrhage in the Sylvian fissure. However, CT did not show brain edema (Figure 1). Subsequently, the patient underwent intracranial pressure sensor insertion. The initial increased intracranial pressure (ICP) was 4 mmHg. Additionally, she was administered midazolam for sedation, levetiracetam for seizure prophylaxis, and mannitol to control ICP and was supported by a ventilator.

**Figure 2:**
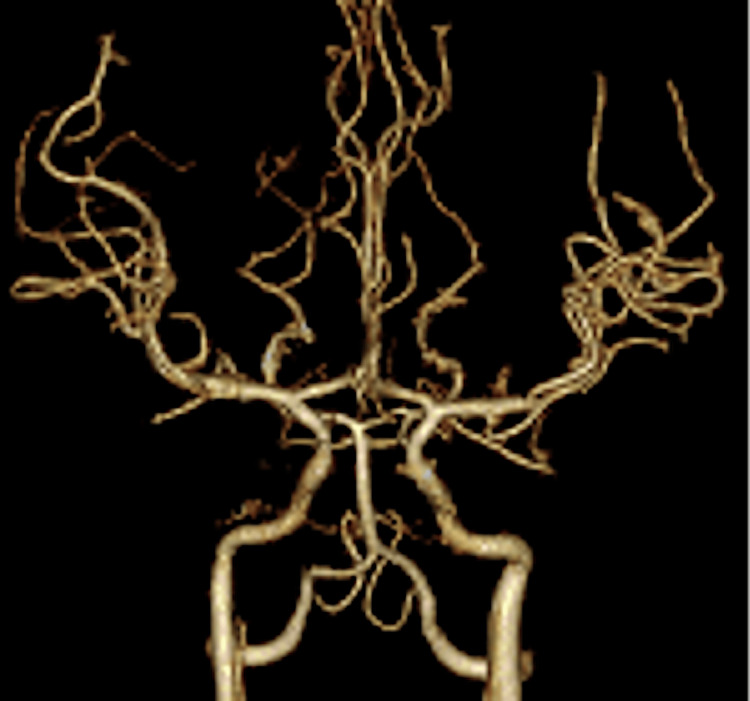
A CT angiography on the second day from the onset did not show any suspicious regions of cerebral vasospasm nor brain edema. CT: computed tomography

**Figure 3:**
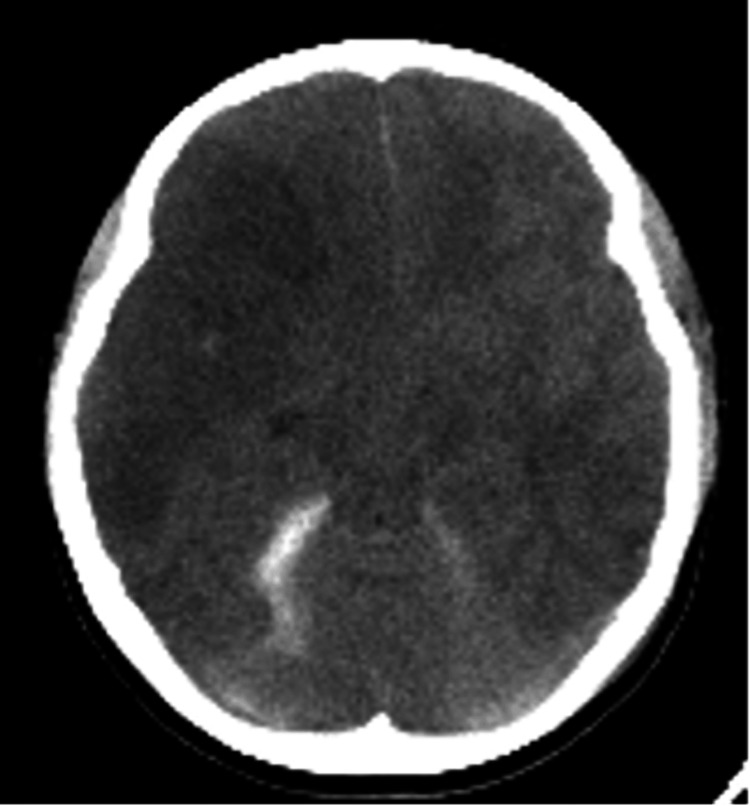
A CT scan of the head on the seventh day from the onset showed extensive ischemic lesions in the right middle cerebral artery territory and left rectal gyrus. CT: computed tomography

**Figure 4:**
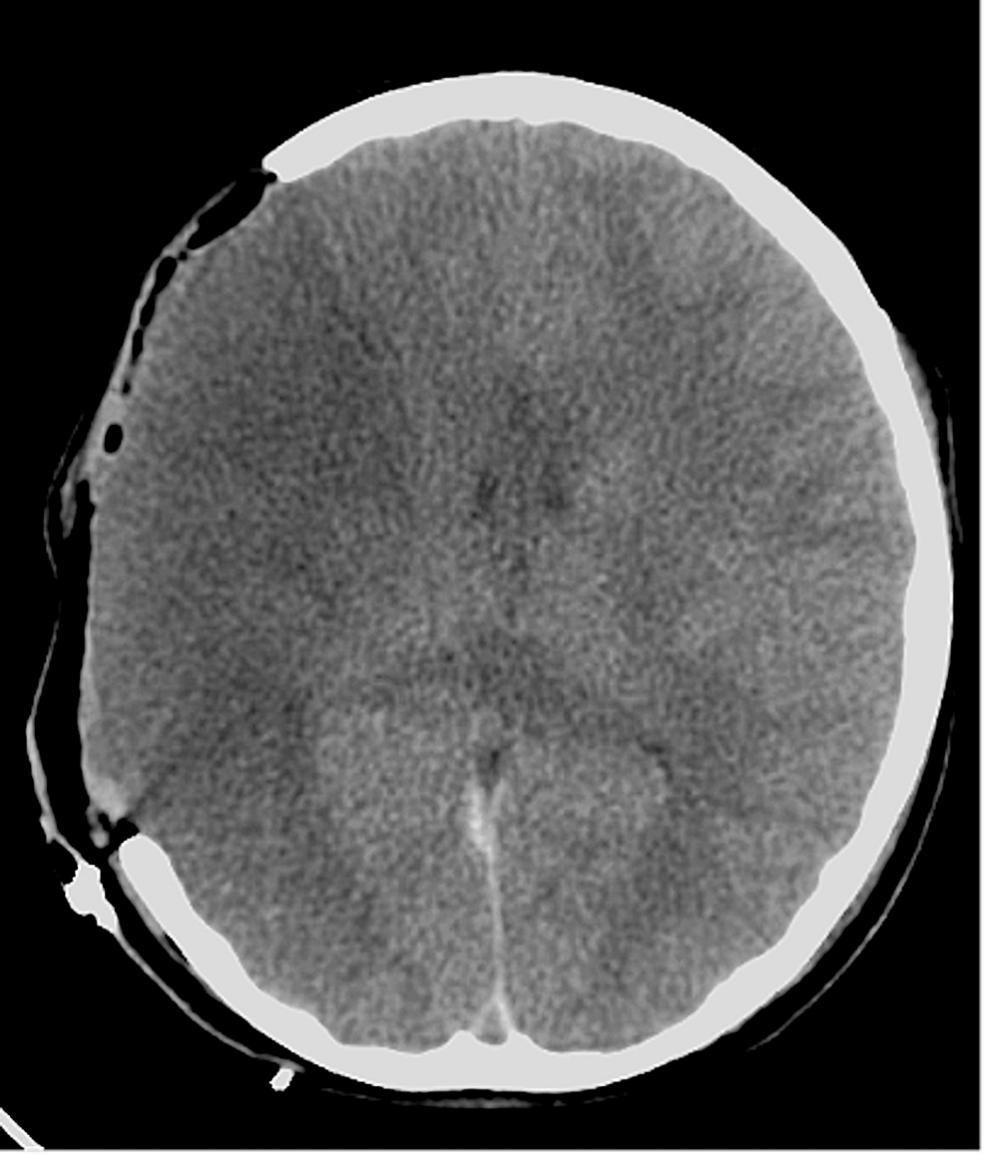
On the seventh day from the onset, we performed decompressive craniectomy to reduce the patient’s ICP. ICP: intracranial pressure

**Figure 5:**
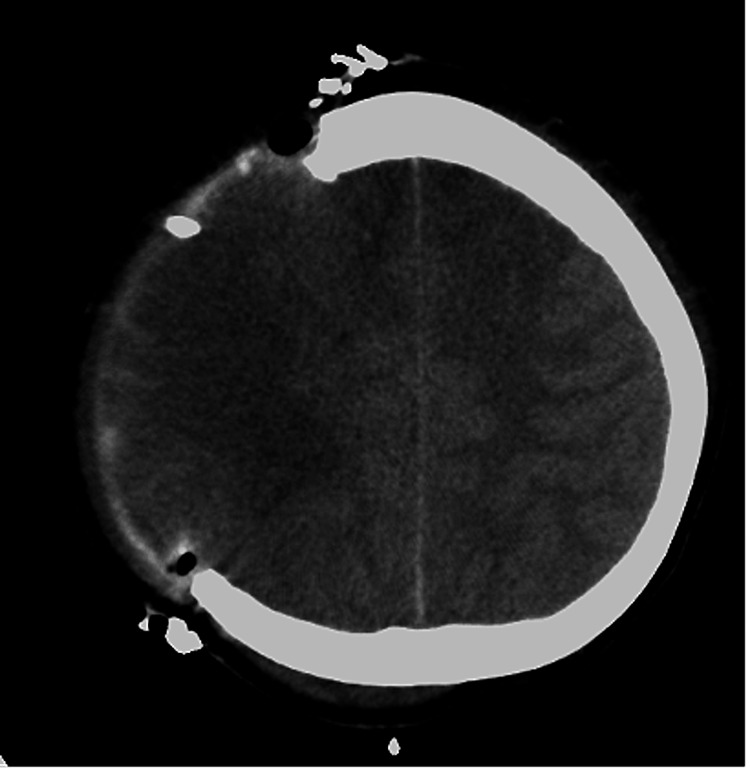
A CT scan of the head on the eighth day from onset showed an ischemic lesion in the left anterior cerebral artery territory. CT: computed tomography

**Figure 6:**
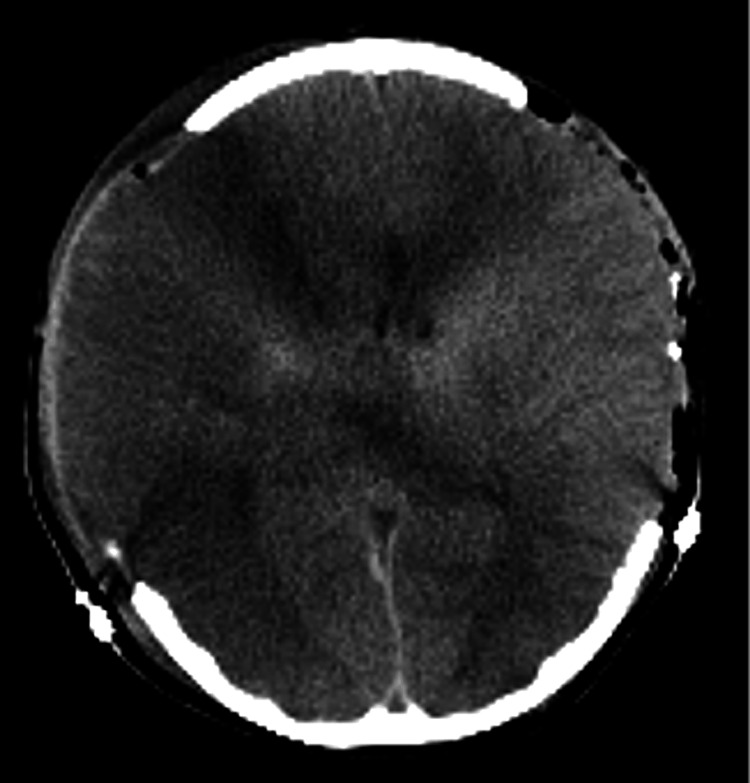
On the eighth day from onset, we added external decompressive craniectomy on the left side.

**Figure 7:**
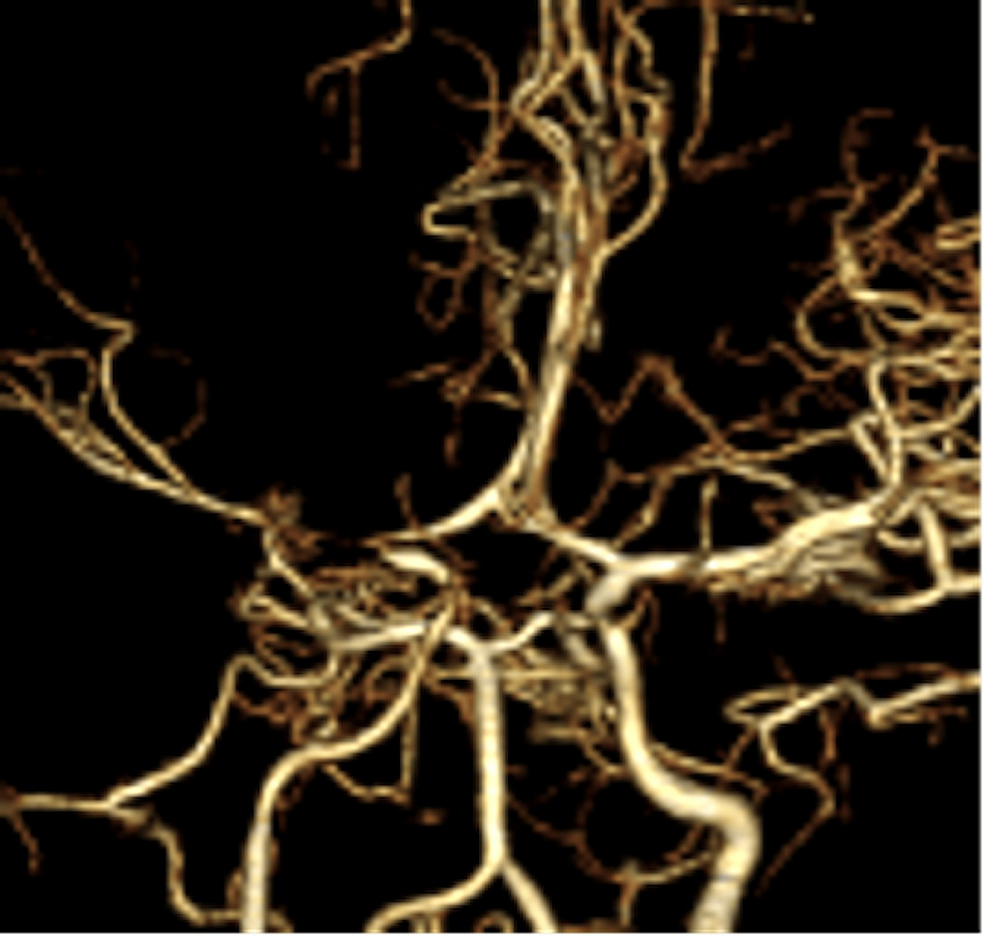
A CT angiography scan on the ninth day showed severe MCA stenosis, suspicious for cerebral vasospasm. CT, computed tomography; MCA, middle cerebral artery

**Figure 8:**
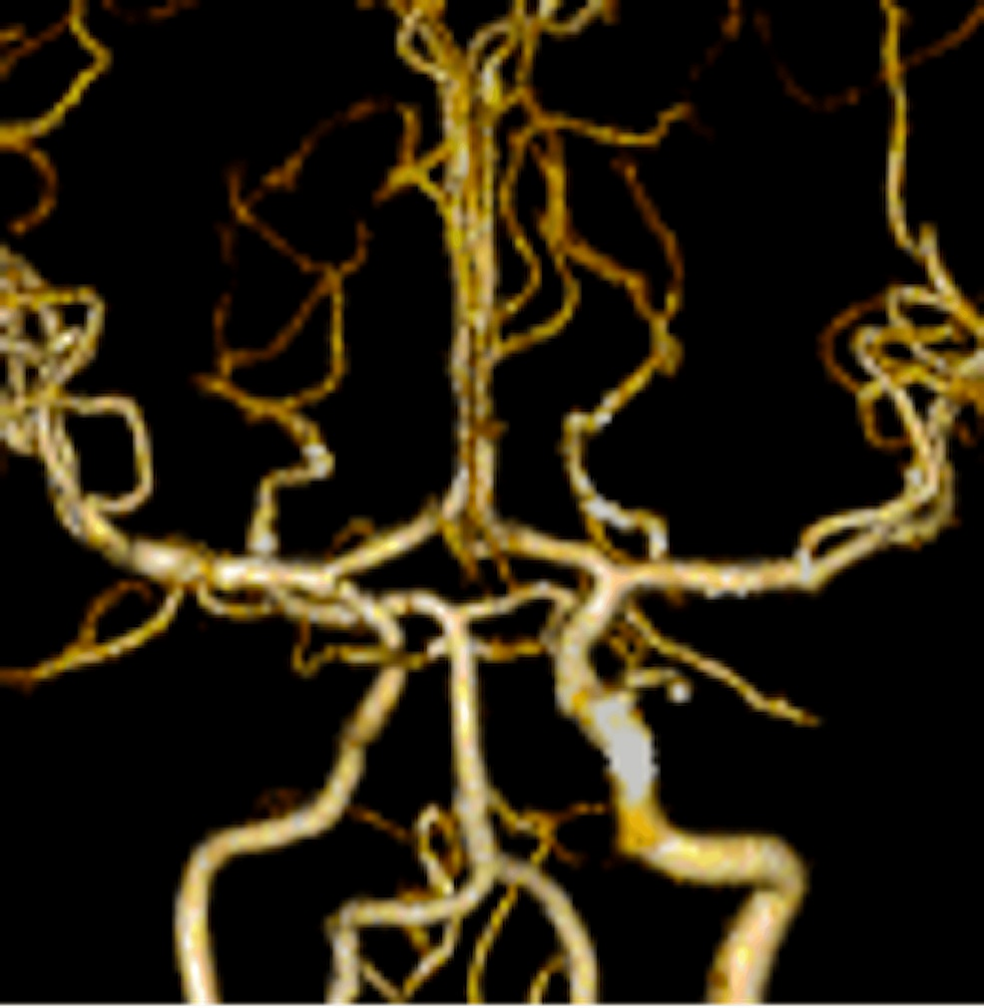
A CT angiography on the 29th day showed an almost normal shape of MCA. CT, computed tomography; MCA, middle cerebral artery

On the second day, CT angiography (CTA) revealed no signs of cerebral vasospasm nor brain edema (Figure 2). On the fourth day, CT showed mild brain edema without increasing ICP. However, her ICP increased to 40 mmHg on the seventh day. A CT scan of the head demonstrated cerebral infarction in the right middle cerebral artery (MCA) region (Figure 3). Therefore, we performed decompressive craniectomy to reduce ICP (Figure 4). On the eighth day, CT of the head showed a low-density area in the left anterior cerebral artery (ACA) region (Figure 5). Consequently, external decompression was performed on the left side (Figure 6). Postoperative computed tomography angiography (CTA) indicated severe stenosis due to right MCA vasospasm (Figure 7). CTA on the 29th day demonstrated the recovery of MCA vasospasm (Figure 8). On the 31st day, we performed cranioplasty. Subsequently, the patient demonstrated significant improvement and was able to write her name and stand with the assistance of a brace. Finally, she was transferred to another hospital for rehabilitation.

## Discussion

Epidemiology

The pathophysiology of cerebral vasospasm following head trauma remains controversial. In subarachnoid hemorrhage, spasmodic and neuroinflammatory substances from subarachnoid blood promote cerebral vasospasm [7]. However, cerebral vasospasm can be induced without subarachnoid hemorrhage [8]. The pathophysiology of post-traumatic cerebral vasospasm is suggested to involve mechanical stretch, inflammation, calcium dysregulation, endothelin, contractile proteins, cerebral metabolic products, and cortical spreading depolarization [8]. Additionally, polymorphisms in the apolipoprotein E gene have been reported to be associated with post-traumatic cerebral vasospasm [9].

The risk factors for post-traumatic cerebral vasospasm are a low GCS score of <9 on admission and young age (<30 years) [10]. In a study of 69 children, the incidence rate of cerebral vasospasm was 8.5% in moderate traumatic brain injury (GCS: 9-12) and 33.5% in severe traumatic brain injury (GCS: 8 or less) [3].

Post-traumatic cerebral vasospasm typically develops within two to three days of the initial injury. However, it sometimes occurs after the sixth day [4]. This time window makes the diagnosis of cerebral vasospasm difficult. Post-traumatic cerebral vasospasm deteriorates neurological outcomes [3]. Therefore, diagnostic modalities for early detection should be considered in patients with risk factors (e.g., low GCS and young age).

Secondary brain injury is induced by the release of excitatory neurotransmitters, intracellular calcium, free radicals, and cytokines [11]. These biochemical events lead to additional cellular damage and contribute to the development of angiogenic cerebral edema, which disrupts the blood-brain barrier and causes fluid leakage from capillaries, particularly affecting the white matter [12]. In the present case, brain swelling following post-traumatic head injury due to the development of angiogenic cerebral edema led to an increase in ICP (40 mmHg) and a subsequent decrease in cerebral perfusion pressure (CPP) (30 mmHg). This may have contributed to the extensive infarction in the right MCA region.

Treatment

The purpose of treatment for vasospasm is to prevent ischemic strokes.

The administration of substances such as magnesium sulfate, statins, fasudil hydrochloride, erythropoietin, endothelin-1 antagonists, nitric oxide progenitors, and sildenafil is currently used to treat cerebral vasospasm [13].

Surgical interventions, such as balloon angioplasty; intravascular stent placement; and the intra-arterial administration of vasodilators such as papaverine, verapamil, and nicardipine should be considered when medical therapy is ineffective [14,15]. Additionally, a superficial temporal artery-to-middle cerebral artery (STA-MCA) bypass is considered when cerebral blood flow decreases due to the progression of stenosis or infarcted areas [16].

In our case, the patient underwent decompressive craniectomy for ischemic stroke. Extensive ischemia in the right MCA territory resulted in significant cerebral edema, leading to the physical compression of ACA. Consequently, induced ischemia also affected the left ACA. Therefore, we added decompressive craniectomy to the left side on the eighth day after onset. Prophylactic antithrombotic treatment is controversial. In addition, the initial CT scan showed an intracranial hemorrhage, so we hesitated to initiate it. On the other hand, if cerebral vasospasm can be diagnosed early, antithrombotic and anti-vasospastic therapies should be initiated. Therefore, we recommend the consideration of performing or further investigation into more frequent use of CTA, MRA, or digital subtraction angiography (DSA) for two weeks to diagnose cerebral vasospasm.

## Conclusions

We encountered a pediatric case of cerebral infarction due to post-traumatic cerebral vasospasm. In cases of trauma, if no abnormalities are detected during the initial cerebrovascular evaluation, serial evaluations are typically not conducted. In our case, the patient presented with low GCS from the beginning and was under sedation, masking neurological symptoms. Therefore, we could only detect the neurological event by increased ICP. Our study suggests that physicians caring for high-risk patients consider the use of vascular imaging modalities, especially in the acute phase, to detect cerebral vasospasm in young head trauma patients with severely depressed consciousness and high-risk factors.
